# The association between serum folate and gestational diabetes mellitus: a large retrospective cohort study in Chinese population

**DOI:** 10.1017/S136898002200194X

**Published:** 2023-05

**Authors:** Xiao-Hui Liu, Zhi-Juan Cao, Li-Wei Chen, Dong-Lan Zhang, Xiao-Xian Qu, Yu-Hong Li, Yu-Ping Tang, Yi-Rong Bao, Hao Ying

**Affiliations:** 1 Department of Obstetrics, Shanghai Key Laboratory of Maternal Fetal Medicine, Shanghai Institute of Maternal-Fetal Medicine and Gynecologic Oncology, Shanghai First Maternity and Infant Hospital, School of Medicine, Tongji University, No. 550 Hunan RD, Shanghai 201204, People’s Republic of China; 2 Department of Clinical Research Center, Shanghai Key Laboratory of Maternal Fetal Medicine, Shanghai Institute of Maternal-Fetal Medicine and Gynecologic Oncology, Shanghai First Maternity and Infant Hospital, School of Medicine, Tongji University, Shanghai, People’s Republic of China; 3 Department of Epidemiology, Fielding School of Public Health, University of California at Los Angeles, Los Angeles, CA, USA; 4 Division of Health Services Research, Department of Foundations of Medicine, New York University, Long Island, School of Medicine, Mineola, NY, USA

**Keywords:** Folate/folic acid, Vitamin B_12_, Gestational diabetes mellitus, Cohort, Chinese

## Abstract

**Objective:**

To investigate the association between folate levels and the risk of gestational diabetes mellitus (GDM) risk during the whole pregnancy.

**Design:**

In this retrospective cohort study of pregnant women, serum folate levels were measured before 24 gestational weeks (GW). GDM was diagnosed between 24th and 28th GW based on the criteria of the International Association of Diabetes and Pregnancy Study Groups. General linear models were performed to examine the association of serum folate with plasma glucose (i.e. linear regressions) and risk of GDM (i.e. log-binomial regressions) after controlling for confounders. Restricted cubic spline regression was conducted to test the dosage–response relationship between serum folate and the risk of GDM.

**Setting:**

A sigle, urban hospital in Shanghai, China.

**Participants:**

A total of 42 478 women who received antenatal care from April 2013 to March 2017 were included.

**Results:**

Consistent positive associations were observed between serum folate and plasma glucose levels (fasting, 1-h, 2-h). The adjusted relative risks (RR) and 95 % CI of GDM across serum folate quartiles were 1·00 (reference), 1·15 (95 % CI (1·04, 1·26)), 1·40 (95 % CI (1·27, 1·54)) and 1·54 (95 % CI (1·40, 1·69)), respectively (*P*-for-trend < 0·001). The positive association between serum folate and GDM remained when stratified by vitamin B_12_ (adequate *v*. deficient groups) and the GW of serum folate measurement (≤13 GW *v*. >13 GWs)

**Conclusions:**

The findings of this study may provide important evidence for the public health and clinical guidelines of pregnancy folate supplementation in terms of GDM prevention.

Folate, a B vitamin, serves as a cofactor and coenzyme in various physiologic processes, including genome maintenance and repair, gene expression regulation, amino acid metabolism, neurotransmitter synthesis and myelin formation^(1,2)^. Unable to synthesise folate, human beings depend on dietary intake and/or supplementation to meet the folate requirement for maintaining normal biological functions^(3)^. The metabolic demand for folate increases during pregnancy due to rapid maternal and fetal cellular growth and development^(4)^.

Periconceptional folate deficiency has been linked with increased risks of neural tube defects^(3)^. Strong evidence from different countries has shown that periconceptional supplementation of folic acid (FA) dramatically reduced the risk of pregnancies complicated with neural tube defects^(5,6)^. Based on these findings, public health agencies worldwide issued recommendations advising women to take FA supplements at a dose of 400 μg/d for 4–12 weeks before pregnancy and 8–12 weeks during early pregnancy^(7,8)^.

Nevertheless, most women take FA for longer periods than recommended. Many continue taking FA as a prenatal vitamin and mineral supplement throughout pregnancy^(9)^. For instance, a study from the Boston Birth Cohort^(10)^ showed that over half of the mothers took FA supplementation almost daily during the second and third trimesters, resulting in a wide range of maternal plasma folate levels approximately 23 % elevated (>45·3 nmol/l). Another recent survey from China reported that 30·7 % of women took FA for 12–24 weeks during pregnancy^(11,12)^.

Although FA supplementation was considered safe for pregnant women^(13)^, concerns about its possible adverse effects on other pregnancy and birth outcomes are rising. For example, a recent study from the Shanghai Preconception Cohort with 1058 participants showed that higher maternal erythrocyte folate levels were positively associated with the risk of gestational diabetes mellitus (GDM) in early pregnancy^(14)^. Affecting approximately 5 % to 17 % of all pregnancies worldwide^(15)^, GDM is a major gestational complication associated with short- and long-term adverse outcomes for both mothers and neonates^(16,17)^. However, studies focusing on the folate levels of the first and second trimesters are scarce.

The neural tube normally closes 24–28 d after conception, while other major malformations develop within 12 weeks of gestation^(13)^, after which time there is no proven benefit of taking FA^(14)^. Compared with the first trimester, it is all the more essential to weigh the pros and cons of FA supplements in the subsequent trimesters. Based on the above findings, this study examined the association between serum folate and GDM risk during the first and second trimesters in a large Chinese cohort.

## Methods

### Research design and subjects

The participants were selected from a cohort derived from the electronic medical records of pregnant women (*n* 43 846) who received prenatal care at a tertiary level A grade hospital in Shanghai, China, between April 2013 and March 2017. Women without serum folate data before the 24th week of gestation (GW; *n* 1368) were excluded, and a total of 42 478 women with live births and no glucose levels suggestive of type 2 diabetes were included in this study. At their first prenatal visit, all participants provided informed consent to use their electronic medical record in future research. This study was approved by the Human Ethics Committee of the Hospital in October 2017.

### Assessment of sociodemographic variables

As a routine process, all participants were required to complete a questionnaire and undergo a blood test at their first prenatal visits. Maternal age, education, pre-pregnancy weight and height were self-reported and collected through the questionnaire. Data on parity, fertilisation method, fetal gender and the number of fetuses (singleton or twins) were extracted from the electronic medical records of the Hospital Information System.

### Assessment of serum folate and vitamin B_12_

As a part of the routine blood test in their first prenatal visits to identify vitamin deficiencies, serum folate and vitamin B_12_ levels were measured using the chemiluminescence method in an ARCHITECT i2000SR immunoassay analyzer (Beckman LX20 Pro analyzer, Beckman Coulter). The inter-assay CV were 3·4 % to 9·6 %, and the intra-assay CV were 2·0 % to 3·6 % for serum folate (normal range: 13·4 to 56·2 nmol/l). Serum folate and vitamin B_12_ levels were extracted from the Laboratory Information System.

### GDM diagnosis

GDM was screened using a one-step method and diagnosed according to the International Association of Diabetes and Pregnancy Study Groups (IADPSG) criteria^(18)^. Briefly, all participants underwent a 75-g oral glucose tolerance test between the 24th and 28th GW. Participants with fasting plasma glucose ≥ 5·1 mmol/l, 1-h plasma glucose (1-h PG) ≥ 10 mmol/l or 2-h plasma glucose (2-h PG) ≥ 8·5 mmol/l after the oral glucose tolerance test tests were diagnosed as GDM.

### Statistical analysis

For the lack of a specified cut-off for folate levels during pregnancy, serum folate levels were typically categorised into two groups according to the upper level of optimal serum folate (45·3 nmol/l) among women of reproductive age. We presented the descriptive data as frequencies, *n* (%), for categorical variables and mean (sd) for continuous variables. To compare the baseline characteristics between the excessive folate group (>45·3 nmol/l) and the normal folate group (≤ 45·3 nmol/l), we conducted the Student’s *t*-test or the Kolmogorov–Smirnov test (variables with skewed distribution) for continuous variables and Pearson’s Chi-squared test for categorical variables.

The main exposure, serum folate, was treated as both continuous and categorical variables (quartiles). Linear regression models were performed to analyse the association between serum folate (per sd) and plasma glucose (fasting, 1-h, 2-h) in oral glucose tolerance test. Log-binomial regression models were conducted to estimate the relative risks (RR) for GDM. The 95 % CI were adopted for serum folate (quartiles). We excluded *in vitro* fertilisation (IVF; *n* 1731) or multiple pregnancies (twins; *n* 860) from the primary analysis and adopted the following potential confounders for the multivariate models: BMI status (normal: BMI < 24·0 kg/m^2^; overweight or obesity: BMI ≥ 24·0 kg/m^2^), fetal gender, parity, vitamin B_12_, maternal age and maternal education. The significance of the linear trends across quartiles of serum folate levels was evaluated using the median value for each quartile and analysed as a continuous variable in the multivariate models.

We also conducted several sensitivity analyses. First, we stratified our analyses by serum vitamin B_12_ levels (< 148 *v*. ≥ 148 pmol/l) or the trimester when serum folate was measured (≤13 *v*. >13 GW). Second, we used Rubin’s causal model^(19)^ to quantify the robustness of causal inferences and interpret how much the bias must be to invalidate an inference in terms of replacing observed cases with counterfactual cases or cases from an unsampled population^(19)^. Third, we assessed the folate-GDM association by including IVF and multiple pregnancies. Additionally, restricted cubic spline regressions were performed with five knots (P_5_, P_25_, P_50_, P_75_ and P_95_) to investigate whether there was a threshold between serum folate and GDM risk.

Statistical analyses were performed on Stata 15.0 (StataCorp., LP), and restricted cubic spline was implemented with R (version 4.0.3). *P* < 0·05 was considered statistically significant.

## Results

Among the 42 478 women, 5122 (12·1 %) developed GDM. The GDM incidence of the excessive serum folate group (13·9 %) was higher than that of the normal serum folate group (10·2 %). Most characteristics, including maternal age, nullipara, IVF, singleton or twins, fetal gender, maternal education, serum vitamin B_12_ level and the GW of folate measurement, were significantly different between the two groups. The three oral glucose tolerance test values, i.e. fasting plasma glucose, 1-h blood glucose and 2-h blood glucose, were significantly higher in the excessive serum folate group than in the normal serum folate group (Table 1).

**Table 1:** Baseline characteristics of study participants between compared groups

Characteristics	All (*n* 42 478)		Normal serum folate (50·6 %)		Excessive serum folate (49·4 %)		*P* *
	*n*	%	*n*	%	*n*	%	
Categorical variable							
GDM							
No	37 356	87·9	19 290	89·8	18 066	86·1	<0·001
Yes	5122	12·1	2204	10·2	2918	13·9	
Maternal age group (years)							
22–28	14 411	33·9	8087	37·6	6324	30·1	<0·001
29–35	24 163	56·9	11 673	54·3	12 490	59·5	
>35	3904	9·2	1734	8·1	2170	10·3	
Nulliparous							
Yes	33 343	78·5	16 115	75·0	17 228	82·1	<0·001
No	9135	21·5	5379	25·0	3756	17·9	
IVF							
Yes	1731	4·1	665	3·1	1066	5·1	<0·001
No	40 747	95·9	20 829	96·9	19 918	94·9	
Singleton or twins							
Singleton	41 618	98·0	21 130	98·3	20 488	97·6	<0·001
Twins	860	2·0	364	1·7	496	2·4	
Fetal sex							
Male	21 990	51·8	10 986	51·1	11 004	52·4	0·006
Female	20 488	48·2	10 508	48·9	9980	47·6	
Education(missing = 4362)							
High school or less	2879	7·6	1925	9·8	954	5·2	<0·001
Undergraduate	32 914	86·3	16 673	84·5	16 241	88·3	
Postgraduate	2323	6·1	1127	5·7	1196	6·5	
Pre-pregnancy BMI status							
Normal weight	33 949	79·9	17 145	79·8	16 804	80·15	0·420
Overweight or obese	8529	20·1	4349	20·2	4180	19·9	
Serum vitamin B_12_ (pmol/l)							
Adequate (≥148)	38 161	89·8	18 366	85·5	19 795	94·3	<0·001
Deficient (<148)	4317	10·2	3128	14·5	1189	5·7	
The GW of folate measurement							
≤13GW	1743	4·1	659	3·1	1084	5·2	<0·001
>13GW	40 735	95·9	20 835	96·9	19 900	94·8	
Continuous variable							
	Mean	sd	Mean	sd	Mean	sd	
Maternal age (years)	30·30	3·71	29·96	3·74	30·65	3·64	<0·001
Serum folate (nmol/l)	42·29	12·70	31·73	8·92	53·10	3·69	<0·001
Serum vitamin B_12_ (pmol/l)	355·46	138·83	323·81	127·21	387·89	142·70	<0·001
Fasting plasma glucose (mmol/l)	4·41	0·36	4·39	0·42	4·40	0·42	0·0174
1 h blood glucose (mmol/l)	7·56	1·56	7·40	1·58	7·68	1·62	<0·001
2 h blood glucose (mmol/l)	6·56	1·28	6·42	1·27	6·66	1·35	<0·001
Pre-pregnancy BMI (kg/m^2^)	21·90	2·87	21·92	2·89	21·88	2·85	0·1888
The GW of folate measurement	15·71	2·06	15·96	2·19	15·45	1·88	<0·001

GDM, gestational diabetes mellitus; IVF, *in vitro* fertilisation; GW, gestational weeks.

* Comparation between normal serum folate group (<=45·3 nmol/l) and excessive serum folate group (>45·3 nmol/l).

The linear associations between serum folate and plasma glucose levels are shown in Table 2. Folate levels were positively associated with fasting, 1-h and 2-h plasma glucose levels under different pre-pregnancy BMI status, fetal gender, parity, maternal age and maternal education regardless of vitamin B_12_ status and the GW of folate measurement. The associations between serum folate and 1-h plasma glucose levels were the most obvious, while associations between serum folate and fasting plasma glucose levels were the least significant.

**Table 2:** The associations between serum folate and plasma glucose concentrations

All women (*n* 40 747)†	Plasma glucose					
	Fasting		1 h		2 h	
	Median	IQR	Median	IQR	Median	IQR
mmol/l	4·40	4·20, 4·59	7·50	6·40, 8·60	6·40	5·70, 7·30
	*β*	95 % CI†	*β*	95 % CI†	*β*	95 % CI†
Crude model	0·00	-0·00, 0·01	0·17	0·15, 0·18**	0·14	0·12, 0·15**
	*β*	95 % CI‡	*β*	95 % CI‡	*β*	95 % CI‡
Adjusted model	0·01	0·00, 0·01*	0·15	0·13, 0·17**	0·12	0·11, 0·13**
	*β*	95 % CI	*β*	95 % CI	*β*	95 % CI
By vitamin B_12_ §						
B_12_ deficiency (<148 pmol/l)	0·02	0·01, 0·03*	0·17	0·13, 0·22**	0·13	0·10, 0·17**
B_12_ adequate (≥148 pmol/l)	0·01	0·00, 0·01**	0·14	0·12, 0·15**	0·11	0·10, 0·13**
By the GW of folate measurement§						
≤13GW	0·01	-0·01, 0·04	0·13	0·02, 0·23*	0·16	0·07, 0·24**
>13GW	0·01	0·00, 0·01*	0·15	0·13, 0·17**	0·12	0·11, 0·13**

IVF, *in vitro* fertilisation; GW, gestational weeks.

* *P* < 0·05.

† IVF(*n* 1731), twins (*n* 378) and IVF and twins (482) were excluded; *β* and 95 % CI were calculated with the use of general liner model and was unadjusted, per sd serum folate increment.

‡ Adjusted for BMI status before pregnancy, fetal gender, vitamin B_12_, parity, maternal age and education, per sd serum folate increment.

§ Analysed in the adjusted model, per sd serum folate increment.

** *P* < 0·001.

The median serum folate level by quartiles (nmol/l) was 25·08, 38·82, 50·37 and 56·30, respectively. We found a positive association between maternal serum folate levels and GDM risk (Table 3). The crude incidence of GDM was 9·35 %, 10·75 %, 12·70 % and 14·35 % among women in quartile 1 (lowest level), 2, 3 and 4 groups, respectively. Compared to women with the lowest serum folate levels (Q1, reference), the unadjusted RR of GDM was 1·17 (95 % CI (1·06, 1·28)) in Q2, 1·41 (95 % CI (1·29, 1·54)) in Q3 and 1·62 (95 % CI (1·49, 1·77)) in Q4 (*P*-trend < 0·001). The positive association was persistent and the trend remained statistically significant after adjusting for pre-pregnancy BMI status, fetal gender, parity, maternal age, vitamin B_12_ level and maternal education: the adjusted RR across serum folate quartiles were 1·00 (reference), 1·15 (95 % CI (1·04, 1·26)), 1·40 (95 % CI (1·27, 1·54)) and 1·54 (95 % CI (1·40, 1·69)), respectively (*P*-trend < 0·001).

**Table 3:** The RR and 95 % CI of GDM in relation to maternal serum folate (*n* 40 747)

All women	Serum folate										*P*-trend†
	Q1		Q2		Q3		Q4		Continuous*		
	Median	IQR	Median	IQR	Median	IQR	Median	IQR	Median	IQR	
Serum folate, nmol/l	25·08	19·98, 29·01	38·82	35·68, 41·77	50·37	46·90, 52·66	56·30	54·48, 57·20	44·79	32·46, 54·03	
	%		%		%		%		%		
GDM incidence	9·35		10·75		12·70		14·35		11·78		
	RR	95 % CI‡	RR	95 % CI‡	RR	95 % CI‡	RR	95 % CI‡	RR	95 % CI‡	
Crude model	1·00	Ref	1·17	1·06, 1·28	1·41	1·29, 1·54	1·62	1·49, 1·77	1·18	1·15, 1·21	<0·001
	RR	95 % CI§	RR	95 % CI§	RR	95 % CI§	RR	95 % CI§	RR	95 % CI§	
Adjusted model	1·00	Ref	1·15	1·04, 1·26	1·40	1·27, 1·54	1·54	1·40, 1·69	1·16	1·13, 1·19	<0·001
	RR	95 % CI	RR	95 % CI	RR	95 % CI	RR	95 % CI	RR	95 % CI	
By vitamin B_12_ §											
B_12_ deficiency (<148 pmol/l)	1·00	Ref	1·32	1·02, 1·72	1·22	1·29, 2·30	1·62	1·18, 2·22	1·21	1·10, 1·32	<0·001
B_12_ adequate (≥148 pmol/l)	1·00	Ref	1·11	1·00, 1·23	1·35	1·21, 1·49	1·50	1·35, 1·66	1·15	1·12, 1·19	<0·001
By the GW of folate measurement§											
≤13GW	1·00	Ref	1·52	0·82, 2·82	2·29	1·26, 4·15	2·02	1·14, 3·59	1·24	1·06, 1·46	0·001
>13GW	1·00	Ref	1·14	1·03, 1·26	1·38	1·25, 1·52	1·52	1·38, 1·68	1·16	1·12, 1·19	<0·001

RR, relative risk; GDM, gestational diabetes mellitus; GW, gestational weeks.

* Per sd serum folate increment.

† Test for trend based on variable containing median value for each quartile. IVF(*n* 1731), twins (*n* 378) and IVF and twins (482) were excluded.

‡ RR and 95 %CI were calculated with the use of general liner model. Crude model was unadjusted.

§ Adjusted for BMI status before pregnancy, fetal gender, parity, maternal age, vitamin B_12_ (continuous) and education.

As shown in Table 3, the positive association between serum folate and GDM remained when we stratified analyses by vitamin B_12_ (adequate *v*. deficient groups) and the GW of serum folate measurement (≤13 GW *v*. >13 GW). Rubin’s causal model results showed that to invalidate the inference, 81·12 % of the estimate would have to be due to bias or 30 920 cases would have to be replaced by cases with zero effect. Sensitivity analyses with IVF (*n* 1731) and multiple pregnancies (*n* 860) included showed that the association between serum folate and GDM risk remained positive and generally unchanged (RR = 1·19; 95 % CI (1·16, 1·24)).

**Fig. 1:**
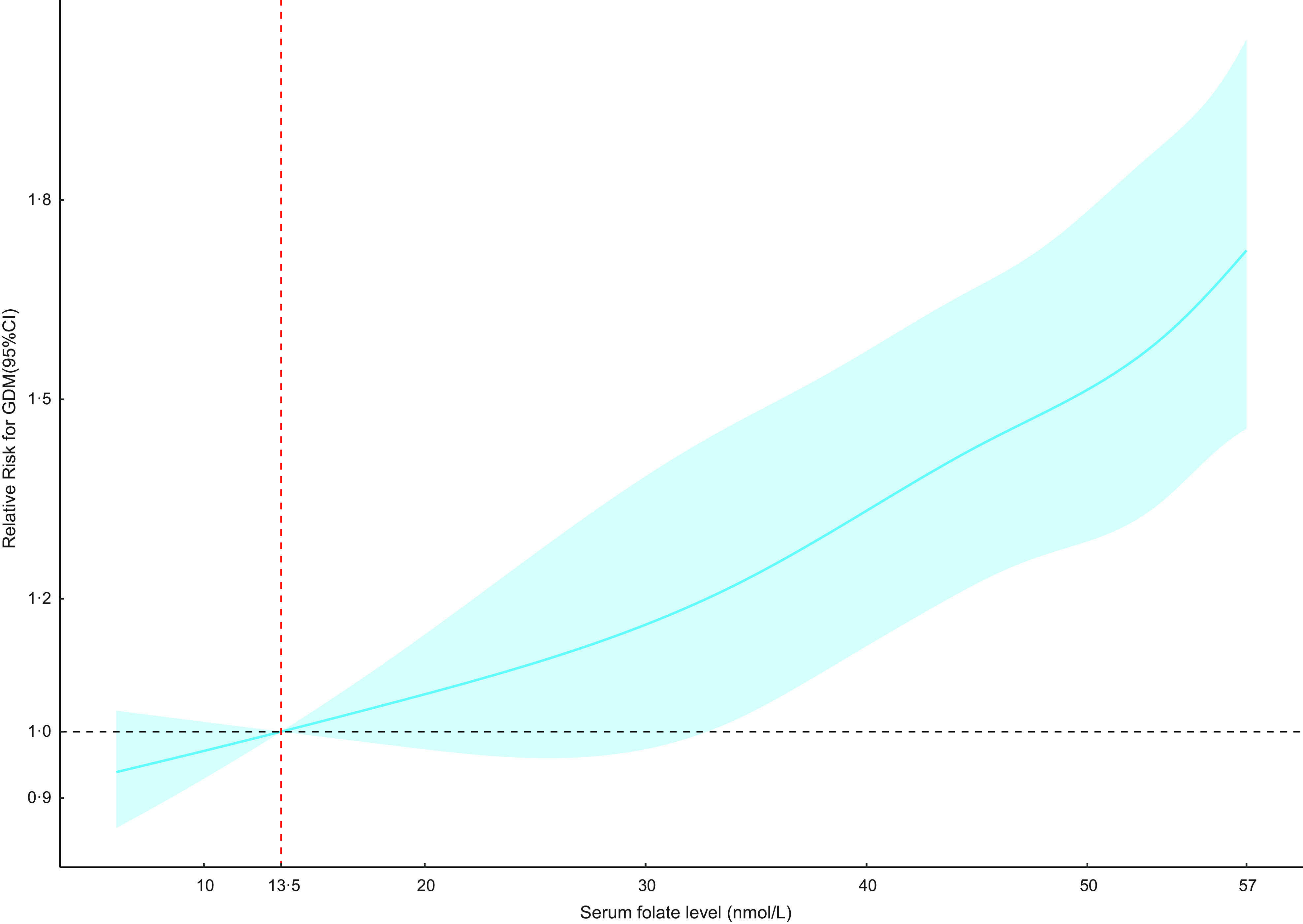
The dose-response relationship between serum folate and RR for GDM. The RR for GDM was estimated in the adjusted model; the solid blue line represented mean values of relative risk for GDM; the blue area represented 95 %CI of the RR for GDM. 13·5 nmol/l was set as the reference. RR, relative risk; GDM, gestational diabetes mellitus

Due to the lack of guidelines for optimal serum folate status among the pregnant population, we adopted guidelines for optimal serum folate status (13·5–45·3 nmol/l) among women of reproductive age instead^(20)^. The restricted cubic spline regression results showed a linear relationship (*P* = 0·808) between serum folate and GDM risk without any threshold (Fig. 1).

## Discussion

In this study on 42 478 Chinese pregnant women, we observed a positive association between serum folate and GDM risk. The RR of GDM increased across serum folate quartiles regardless of vitamin B_12_ status and the GW of folate measurements. The association between serum folate levels and GDM risk was linear without a significant breakpoint or threshold. Sensitivity analysis, stratified analysis and Rubin’s causal model showed good robustness of the estimates.

Although previous cohort studies have associated higher pre-pregnancy supplemental folate intake with lower risks of GDM in American women^(21,22)^, our findings are in line with the results of two Chinese cohort studies recognising positive associations between gestational folate supplementation and GDM risk. A China-Anhui Birth Cohort Study (C-ABCS)^(23)^ associated daily FA supplement consumption in the first trimester with increased risks of GDM (OR = 2·25, 95 % CI (1·35, 3·76)). The Tongji Maternal and Child Health Cohort (TMCHC)^(24)^ revealed a significant correlation between prolonged intake of FA > 800 mg/d and higher risks of GDM (OR = 2·09, 95 % CI (1·30, 3·36)). However, measurements of folate exposure were self-reported (FA supplement) in both of the two studies above. Therefore, we used serum folate instead, which is an objective measure of circulating folate level and could reflect the women’s biological response to both folate from food and supplements. In addition, serum folate mirrored recent folate status and was preferred over erythrocyte-folate as it can be precisely measured using immunological or chromatography methods^(25)^. Our results are also consistent with the results of Shanghai Preconception Cohort^(14)^, which most recently reported that daily folate supplementation in early pregnancy increases the risk of GDM (OR = 1·73, 95 % CI (1·19, 2·53)) and higher maternal erythrocyte folate in early pregnancy is significantly associated with the risk of GDM. In the meantime, this study also extended and supplemented the Shanghai Preconception Cohort study with the association between folate and GDM in the second trimester.

The mechanism through which high serum folate levels cause GDM risks is still unclear. Previous studies mostly focused on the adverse health effects of excessive gestational folate supplementation on neonate^(26)^. Animal experiments indicated that excessive maternal folate supplementation could cause offspring cognitive impairment through the epigenetics pathways^(27)^ and offspring insulin resistance through the mitochondrial damage mechanisms^(28)^. A recent meta-analysis^(29)^ pointed out that the effects of folate supplementation on fasting insulin levels are stronger in women than in men, with similar trends for fasting glucose and the Homeostatic Model Assessment of Insulin Resistance. Granted that these randomised controlled trials are performed in non-pregnant women, they offer very strong evidence against a causal interpretation of the findings presented in this study. The specific mechanism by which excessive gestational folate supplementation affects maternal metabolism has not been reported. The potential effects of folate supplementation on glycaemia warrant investigation in the future.

Although this study revealed a robust association between high folate levels and high GDM risks during the first two trimesters in a large cohort of Chinese pregnant women, it is not without limitations. Firstly, it has been reported that the determinants of folate status may be multiple, including genetic, biological and socio-economic components^(30)^. The polymorphism of genes involved in folate metabolism (e.g. methylene tetrahydrofolate reductase and methionine synthase reductase) is associated with various diseases (cancers, neurological diseases, diabetes, etc.). The epidemiology of the polymorphism of C677T varies with geography and ethnicity, which might contribute to the opposite associations between folate supplementation and GDM in different countries or regions^(21–23)^. However, the interference of polymorphism of genes involved in folate metabolism with the results cannot be ruled out in this study. Therefore, the effects of folate metabolism-related gene polymorphisms should be considered when evaluating the association between folate supplementation and GDM in the future. Secondly, although serum folate levels were measured, supplement-derived folate and dietary-derived folate could not be distinguished. This study also failed to detect unmetabolised folate levels. Studies have associated unmetabolised plasma FA with decreased natural killer cell activity^(4)^, which has also been suggested to be involved in the pathogenesis of GDM. Thus, the potential effects of unmetabolised FA need to be further studied. In addition, as with all retrospective studies, we cannot completely rule out all potential confounding factors, such as gestational weight gain and nutritional status, previous GDM and other socio-economic factors. Only maternal education has been considered. Nevertheless, the result of Rubin’s causal model indicated that the correlation between folate and GDM risk was unlikely to be caused by potential confounding factors. Therefore, the conclusion of this study needs to be further verified by RCT and related mechanism research.

Based on the above, we observed a positive association between serum folate and GDM risk during the first and second trimesters in a large retrospective cohort study of 42 478 Chinese pregnant women. Adverse effects of excessive folate should be considered from a GDM prevention perspective. The optimal threshold of folate level, especially after the first trimester, and the mechanism behind the association warrant further examination.
